# Relationship between TNF-α levels and psychiatric symptoms in first-episode drug-naïve patients with schizophrenia before and after risperidone treatment and in chronic patients

**DOI:** 10.1186/s12888-021-03569-5

**Published:** 2021-11-11

**Authors:** Chen Lin, Ke Chen, Jianjin Yu, Wei Feng, Weihong Fu, Fude Yang, Xiangyang Zhang, Dachun Chen

**Affiliations:** 1 Department of Psychosomatic Medicine, Beijing HuiLongGuan Hospital, Peking University, Beijing, 100096 People’s Republic of China; 2 Beijing HuiLongGuan Hospital, Peking University, Beijing, 100096 People’s Republic of China; 3 Department of Psychiatry and Behavioral Sciences, The University of Texas Health Science Center at Houston, Houston, TX USA

**Keywords:** Schizophrenia, TNF-α, Inflammatory cytokines, PANSS

## Abstract

**Background:**

The influence of antipsychotic drugs on tumor necrosis factor-α (TNF-α) levels is unclear, and there is no consensus on the association between TNF-α and psychotic symptoms. This study aimed to investigate the differences in TNF-α levels and clinical correlations in first-episode drug-naïve (FEDN) patients with schizophrenia before and after treatment and in chronic patients.

**Methods:**

A total of 103 (51 FEDN and 52 chronic) patients and 114 healthy controls were recruited. Demographic and clinical data, including TNF-α levels, were recorded. We used the Positive and Negative Syndrome Scale (PANSS) to measure the psychopathology of all patients.

**Results:**

TNF-α levels before treatment were significantly higher in FEDN patients than in chronic patients and healthy controls. No significant sex differences were found in the TNF-α levels of patients with schizophrenia. The TNF-α levels before treatment were significantly positively related to changes in PANSS negative symptoms in FEDN patients. The TNF-α levels in chronic patients were significantly negatively correlated with the general psychopathology subscales and PANSS total scores.

**Conclusions:**

Increased TNF-α levels in FEDN patients and their correlation with psychopathology indicate that inflammatory cytokines may play a crucial role in the etiopathogenesis of schizophrenia, and inflammation-directed therapy may, therefore, improve negative symptoms.

## Background

Schizophrenia is a complicated and serious mental illness that mostly develops in early adulthood or late adolescence. The point prevalence of global age-standardized for schizophrenia was estimated to be 0.28% in 2016, and global epidemic cases rose from 13.1 million in 1990 to 20.9 million cases in 2016. Globally, schizophrenia leads to the population experiencing 13.4 million years of life living with disability [1]. Despite persistent research, we often fail to achieve the desired outcomes for the treatment of patients with schizophrenia. The pathogenesis of schizophrenia remains unclear; however, neuroinflammation is considered a potential factor [2, 3]. Previous autoimmune diseases raised the risk of schizophrenia by 29%, according to the results of a 30-year population-based registry study [4]. Prenatal maternal infection (including intracellular *Toxoplasma gondii*, influenza, and cytomegalovirus) is associated with schizophrenia in adult children [5]. Nonsteroidal anti-inflammatory drugs can reduce psychotic symptoms of schizophrenia spectrum disorders [6, 7]. The translocator protein (TSPO) is closely related to neuroinflammation. Although there was heterogeneity among studies, such as differences in TSPO radioligands and outcome measurement methods, which led to different results, a recent meta-analysis suggested that TSPO tracers were elevated in the gray matter of patients with schizophrenia when the binding potential was used as the outcome measure, rather than the volume of distribution [8, 9]. Genetic studies have shown that the strongest genetic association of schizophrenia at the population level includes the complement component 4 (C4) allele genes. During the development of schizophrenia, excessive complement activity may help to explain the decrease in synaptic numbers in the brains of patients with schizophrenia [10]. These results indicate a strong link between inflammation and schizophrenia.

TNF-α participates in the process of neuro-immune regulation and autoimmunity, including the regulation of development, repair, signal feedback, differentiation of glial cells during early growth, and maintenance of normal brain morphology [11]. TNF-α signaling is transmitted via two major receptors: TNF-α RI and TNF-α RII. The activation of TNF-α RI has neurodegenerative effects, whereas that of TNF-α RII is neuroprotective [12]. Numerous studies have demonstrated that TNF-α and TNF-α-related signaling pathways may be crucial in the pathophysiological mechanisms of schizophrenia. A study on 2512 patients with schizophrenia indicated that the TNF-α A allele may have an impact on susceptibility to schizophrenia [13]. Several pathways have been found to be highly associated with schizophrenia, including the apoptotic, inflammatory, and immune systems, as well as the TNFR1 pathway [14]. Brain autopsies of patients with schizophrenia showed 2.3-fold increased concentrations of TNF-α protein and mRNA compared to the normal population [15]. A recent analysis of the inflammatory response to an induced microglia-like phenotype (iMG) has shown that cells from patients with schizophrenia have a stronger response to lipopolysaccharide, with TNF-α protein secretion most prominently observed [16]. TNF-α may be an indicator of the immune dysfunction associated with negative symptoms of schizophrenia [17]. Brain TNF-α is the most promising pharmacological target for the treatment of neuroinflammation that is known to be associated with schizophrenia [18].

Although there is discrepancy among several studies, some meta-analyses have shown that patients with first-episode psychosis have higher TNF-α levels than normal controls [19, 20]. However, there were differences in the TNF levels in patients with schizophrenia after treatment. The influence of antipsychotic drugs on the neuroimmune system is unclear, and there is no consensus on whether neuroinflammation is a characteristic marker of schizophrenia or whether it fluctuates with antipsychotic treatments. One meta-analysis demonstrated that the TNF-α levels decrease after antipsychotic treatment [21], while another meta-analysis reported no significant variation in the TNF-α levels pre- and post-treatment, which indicates that TNF-α represents a trait marker [22]. Chronic patients with schizophrenia had lower TNF-α levels than normal controls [23], but another study showed that the TNF-α levels were significantly higher in chronic patients than in healthy controls [24].

There are no consistent results on the relationship between TNF-α and psychiatric symptoms in patients with schizophrenia or on the relationship between changes in the TNF-α levels and psychiatric symptoms. One study found there was no association between TNF-α and psychiatric symptoms at admission and discharge in patients with schizophrenia [25]. However, in patients with chronic schizophrenia, a negative association between the TNF-α levels and psychopathological symptoms has been reported [23]. Few studies have reported the relationship between changes in the TNF-α levels and psychiatric symptoms before and after treatment. Moreover, different antipsychotics have different effects on the levels of inflammatory cytokines. The effects of metabolic syndromes associated with antipsychotics on inflammatory cytokines need to be considered [21]. In addition, male and female immune systems differ in their ability to respond to pathogens, environmental assaults, or autoantigens and their subsequent effects on immune regulation, as well as in the production of inflammatory cytokines after disease stimulation [26]. It is unclear whether sex differences in inflammatory cytokines in patients with schizophrenia exist, and there are few relevant studies, which have also reported inconsistent results. One study showed that female patients have higher interleukin-6 levels, but not TNF-α levels, than male patients [27]. However, other studies found no sex differences in the TNF-α levels [28, 29].

Therefore, the aims of our research were (1) to compare differences in the TNF-α levels among first-episode drug-naïve (FEDN) patients with schizophrenia, chronic patients, and healthy controls; (2) to assess TNF-α changes in FEDN patients before and after treatment with risperidone for 12 weeks and analyze the correlation between the TNF-α levels and psychiatric symptoms in patients with schizophrenia; and (3) to analyze the possible existence of sex differences in the TNF-α levels in patients with schizophrenia.

## Methods

### Participants

Subjects in the schizophrenia group were admitted to the Beijing HuiLongGuan Hospital from January 2015 to December 2018. The healthy subjects were enrolled from the local community in Beijing. After we posted the recruitment information online, all healthy controls that met the inclusion criteria were recruited without any selection. All recruited patients were inpatients. All patients recruited in the study met the diagnostic criteria for schizophrenia spectrum disorders of the Diagnostic and Statistical Manual of Mental Disorders (the fifth edition, DSM-5). All subjects were of Han nationality and aged between 18 and 60 years.

The inclusion criteria for FEDN patients with schizophrenia were as follows: (1) no prescription antipsychotic medication before enrollment; (2) total score ≥ 60 on the Positive and Negative Syndrome Scale (PANSS); and (3) total duration of illness ≤5 years at first onset. The inclusion criteria for chronic patients with schizophrenia were as follows: (1) total duration of illness ≥60 months and (2) steady dosage of oral antipsychotic medication ≥12 months before the start of this study.

The exclusion criteria for patients included (1) severe central nervous system disease, physical illness, immune disease, and recent administration of any immunomodulators; (2) drug or alcohol dependence, illicit drug use such as marijuana in the past 30 days; and (3) pregnancy or lactation. In total, 103 patients were enrolled, including 77 male and 26 female patients, with an average age of 37.65 ± 12.20 years; the mean duration of illness was 154.47 ± 144.65 months, and the average years of education was 10.61 ± 3.14 years.

The inclusion criteria for healthy controls were as follows: (1) 18–60 years old; (2) no medical history of mental diseases, including schizophrenia, bipolar disorder, or substance dependence; no family history of mental illness at present or in the past; and (3) good physical health status, without serious physical diseases, immune diseases, or organic brain diseases, and without recent administration of any immunomodulators. The exclusion criteria for healthy controls were the same as those for the patient group. A total of 114 subjects were recruited, including 21 male and 93 female patients, with a mean age of 44.31 ± 14.40 years; the average years of education was 8.49 ± 3.96 years.

Trained research staff administered a detailed questionnaire to all subjects to collect their sociodemographic data, general information, and medical status. We obtained an entire medical history, physical examination, and laboratory tests from all the participants, excluding those subjects who had abnormal examinations or medical illnesses, as mentioned in the previous exclusion criteria. Our research was approved by the Ethics Committee of Beijing HuiLongGuan Hospital; all enrolled participants and/or their legal guardians signed the informed consent form.

## Methods

### Clinical treatment and symptom assessment

FEDN patients were administered an oral monotherapy of risperidone (4–6 mg) and were prohibited from using other antidepressants and mood stabilizers. Patients with extrapyramidal syndrome could be treated with diphenol hydrochloride. A PANSS assessment was performed to evaluate the psychopathological symptoms of chronic patients and FEDN patients with schizophrenia before and after 12 weeks of treatment with antipsychotics. The PANSS contains a total of 30 items, among which 7 items are related to positive symptoms, 7 items to negative symptoms, and 16 items to general psychopathology. Each item is scored on a scale of 1 (none) to 7 (extremely severe) for psychopathological symptoms. After assessment by two psychiatrists with prior PANSS training, the intra-group correlation coefficient was > 0.80.

### Serum TNF-α detection

On the second day after enrollment, following an overnight fast (avoiding food for > 10 h), blood samples were collected at 6:00–8:00 AM from the forearm veins of the chronic patients and healthy controls, and from the FEDN patients with schizophrenia before and after 12 weeks of antipsychotic treatment. After storage at room temperature for 1 h, the collected serum was centrifuged for 10 min at 3000 r/min and stored in a refrigerator at − 80 °C for examination. TNF-α levels were measured using a commercially available enzyme-linked immunosorbent assay kit (NeoBiosciece Technology, Beijing, China). The same researcher tested the serum samples for all tests. The sensitivity of detection was 4 pg/mL, and the coefficients of variation inter-assay and intra-assay were 9 and 6%, respectively.

#### Statistical methods

SPSS software version 22.0 was used to perform the statistical analyses. We applied the one-sample Kolmogorov-Smirnov test to examine the normality of data. The χ2 test was used to analyze the differences between the categorical variables, and one-way ANOVA or independent sample *t*-tests were performed to compare differences between the groups in terms of their consecutive demographic variables. We used the Kruskal-Wallis H test to analyze differences between the groups that had non-normally distributed variables. The correlation between the TNF-α levels and psychotic symptoms was analyzed using Pearson or Spearman correlations. Nonparametric tests of two related samples were used to compare the TNF-α levels before and after treatment. Multivariable linear regression analysis was used to analyze the correlations between the TNF-α levels and demographic or psychiatric symptoms. We used a generalized linear model to analyze the relationships between non-normally distributed variables and the clinical data. The generalized linear mixed model was used to test the effect of TNF-α on psychopathology before and after treatment. Statistical graphing was performed using GraphPad Prism 7.0 software. We applied the Bonferroni correction to adjust for multiple testing. All *p*-values were two-tailed, with a significance level of < 0.05.

## Results

### Demographic data

As shown in Table 1, significant differences were found in age, sex, and education among the three groups (*p* < 0.001). The results of the post-hoc pairwise comparisons revealed significant differences in sex among the three groups. There were more males than females among the FEDN patients and chronic patients and more females than males among the healthy controls (*p* < 0.05). The age of FEDN patients was significantly lower than that of chronic patients and healthy controls (*p* < 0.001), whereas there was no significant difference in age between chronic patients and healthy controls (*p* > 0.05). The education level of FEDN patients was significantly higher than that of chronic patients and healthy controls (*p* < 0.05), but there was no significant difference in education level between chronic patients and healthy controls (*p* > 0.05). There was no significant difference in the age of onset between FEDN patients with schizophrenia and chronic patients (*p* > 0.05), and significant differences were observed in the duration of illness, PANSS total score, and the three subscale scores between the two groups (*p* < 0.05).

**Table 1:** Demographics of FEDN, chronic patients with schizophrenia and healthy controls

	FEDN patients (*n* = 51)	Chronic patients (*n* = 52)	Healthy controls (*n* = 114)	F or T or Zor X^2^	*P*-value
Sex, male/female	32/19	45/7	21/93	75.229	< 0.001
Age (years)	27.51 ± 8.71	47.6 ± 4.40	44.31 ± 14.40	48.521	< 0.001
Education (years)	11.84 ± 3.57	9.40 ± 2.04	8.49 ± 3.96	16.133	< 0.001
Age of onset (years)	25.63 ± 9.12	23.92 ± 5.94		1.122	0.265
Duration of illness (months)	18 (6,36)	284.08 ± 83.23		−8.749	< 0.001^a^
BMI (kg/m^2^)	22.5 ± 3.80	23.56 ± 4.26		−1.140	0.256
Antipsychotic dose (mg/day) (chlozapine equivalents)		375 (262,500)			
PANSS total score	86.71 ± 20.39	76.81 ± 18.28		2.595	0.011
Positive subscore	26.33 ± 6.13	16.42 ± 6.17		8.175	< 0.001
Negative subscore	18.94 ± 7.49	24.71 ± 6.33		−4.224	< 0.001
General psychopathology subscore	41.43 ± 12.59	35.67 ± 10.12		2.561	0.012
TNF-α (pg/ml)	19 (9.86,34.33)	10.24 ± 2.14	1.98 (0.00,26.34)		< 0.001*

Note: FEDN, first-episode drug-naïve; PANSS, Positive and Negative Syndrome Scale; TNF-α, tumor necrosis factor-α. ^a^ Mann-Whitney U test, * Kruskal-Wallis H test

### Higher TNF-α levels in FEDN patients

**Fig. 1:**
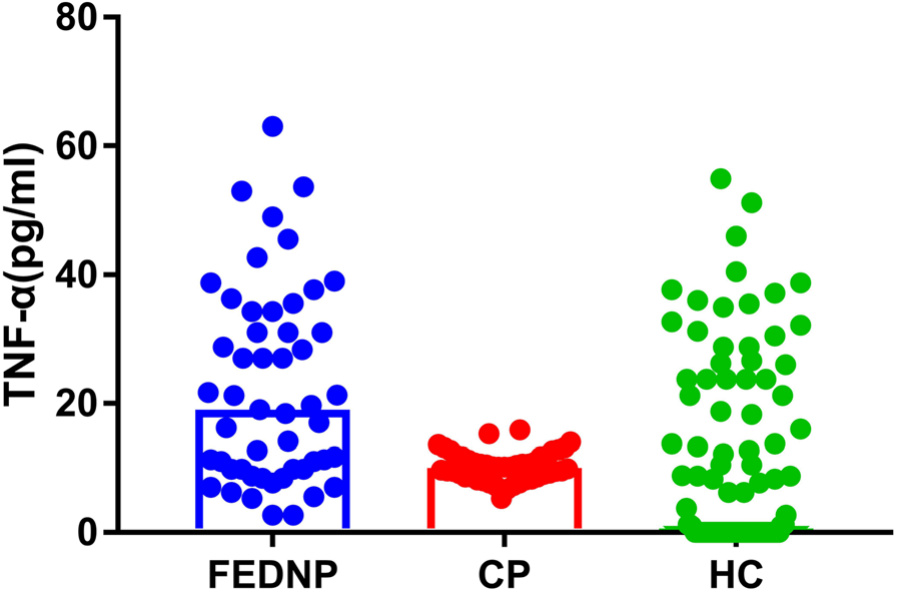
The scattergram of serum TNF-α levels in patients with schizophrenia and healthy controls. This figure presents a scattergram of serum TNF-α levels from FEDN patients with schizophrenia before treatment (FEDNP) (*n* = 51), chronic patients with schizophrenia (CP) (*n* = 52), and healthy controls (HC) (*n* = 114)

First, the normality test showed that the TNF-α levels in FEDN patients before treatment and in normal controls did not conform to a normal distribution. The TNF-α levels in chronic patients were normally distributed. The Kruskal-Wallis H test revealed significant differences in the TNF-α levels between patients with schizophrenia and healthy controls (*p* < 0.001; Table 1 and Fig. 1). Considering that age, sex, and education may be confounding factors, we utilized a generalized linear model using these as covariates. The model still suggested significant differences in the TNF-α levels among the three groups (Wald χ2 = 7.694, *p* < 0.05). Further pairwise comparison analyses showed that the TNF-α levels in FEDN patients before treatment were significantly higher than those in chronic patients and healthy controls (*p* < 0.05). The significant differences in FEDN patients and healthy controls passed the Bonferroni correction (*p* < 0.0167). No significant difference was found in the TNF-α levels between chronic patients and healthy controls (*p* > 0.05). In the pairwise comparisons of the generalized linear model, significant differences were noted in the TNF-α levels among the subjects of different ages, and the TNF-α levels in the lower age group were higher than those in the higher age group (*p* < 0.01). There was no interaction between age and group (*p* > 0.05). There was no significant difference in the TNF-α levels among subjects with different education levels (*p* > 0.05).

### Sex differences in TNF-α levels

To analyze possible sex differences in the TNF-α levels in patients with schizophrenia, we used a stratification analysis, which revealed no significant differences in the TNF-α levels between males and females in the three groups (*p* > 0.05). The generalized linear model was conducted with the TNF-α level as the dependent variable and sex, age, and education levels as the predictor variables for FEDN patients and chronic patients. The result showed no significant differences in the TNF-α levels between males and females (χ2 = 0.690, *p* > 0.05).

### Correlation between TNF-α levels and psychotic symptoms

The Spearman correlation analysis showed no significant associations between the TNF-α levels and psychotic symptoms (PANSS total score, positive subscale score, negative subscale score, and general psychopathology subscale score) in FEDN patients before and after treatment (*p* > 0.05). After controlling for sex, age, and education level, the regression analysis still showed no significant relationship between the TNF-α levels and psychotic symptoms in FEDN patients before and after treatment (*p* > 0.05). The partial correlation analysis showed that the TNF-α levels in chronic patients were significantly negatively correlated with the PANSS total scores (r = − 0.415, *p* < 0.01) and PANSS general psychopathology subscale scores (r = − 0.416, *p* < 0.01) after controlling for age, sex, and education (Table 2). The multivariable linear regression analysis was conducted with psychotic symptoms as the dependent variable and sex, age, body mass index, age of onset, dose of antipsychotics, and TNF-α levels as the independent variables. The results showed that the TNF-α levels were still negatively correlated with PANSS general psychopathology subscale scores in chronic patients (β = − 0.358, *p* < 0.05).

**Table 2:** Correlation between TNF-α and PANSS scores in FEDN patients before and after treatment and chronic patients

		FEDN patients before treatment			FEDN patients after treatment		Chronic patients	
	Beta	T	*P*	Beta	T	*P*	*r*	*P*
PANSS total score	0.13	0.85	0.398	0.06	0.34	0.734	− 0.42	0.003
Positive subscore	0.11	0.77	0.447	−0.03	− 0.21	0.835	− 0.25	0.085
Negative subscore	0.14	0.93	0.360	0.03	0.16	0.877	−0.26	0.075
General psychopathology subscore	0.07	0.46	0.647	0.13	0.70	0.490	−0.41	0.003

Note: FEDN, first-episode drug-naïve; PANSS, Positive and Negative Syndrome Scale; TNF-α, tumor necrosis factor-α

### Changes in TNF-α levels before and after treatment and correlation with psychotic symptoms

**Fig. 2:**
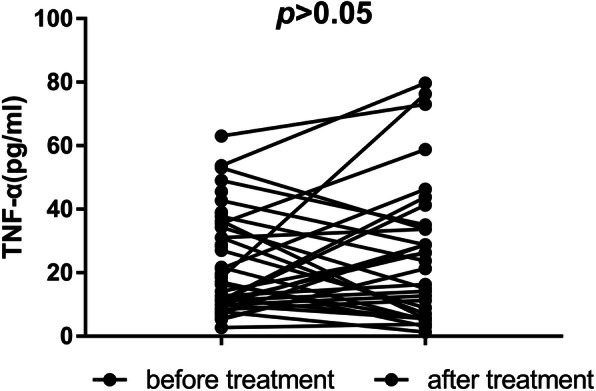
Changes in TNF-α Levels before and after treatment in FEDN patients. The nonparametric Wilcoxon test (for testing two related samples) showed there was no significant difference in the TNF-α levels of FEDN patients before and after 12 weeks of treatment with antipsychotic drugs (Z = -1.477, *p* > 0.05)

**Fig. 3:**
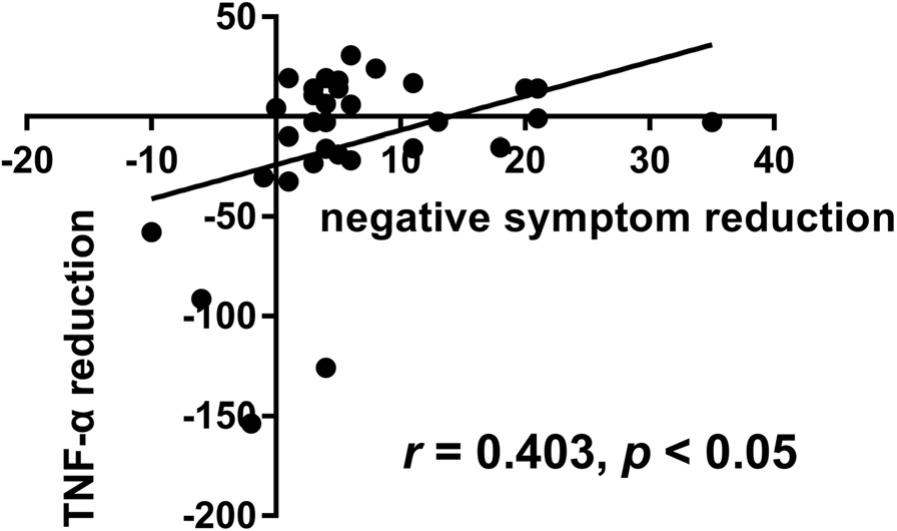
Correlation between the reductions in the TNF-α levels and the PANSS negative subscale reductions in FEDN patients. The Spearman correlation analysis showed that reductions in the TNF-α levels before and after treatment were significantly positively associated with PANSS negative subscale reductions (*r* = 0.403, *p* < 0.05)

The mean TNF-α level in FEDN patients with schizophrenia was 24.95 (7.48, 43.12) pg/mL after treatment. The nonparametric Wilcoxon test (for testing two related samples) was used to compare differences in the TNF-α levels of FEDN patients before and after 12 weeks of treatment with antipsychotic drugs, and there was no significant difference (Z = -1.477, *p* > 0.05; Fig. 2). Considering the seven dropouts during the treatment, we used the generalized linear model to analyze changes in the TNF-α levels before and after treatment, while controlling for age, sex, and education level. The result still showed no significant difference between the TNF-α levels after and before treatment (Wald χ2 = 1.501, *p* > 0.05). The Spearman correlation analysis showed that reductions in the TNF-α levels before and after treatment were significantly positively associated with PANSS negative symptoms before treatment (*r* = 0.507, *p* < 0.01) and positively correlated with PANSS negative subscale reductions (*r* = 0.403, *p* < 0.05; Fig. 3). Then we performed a sensitive analysis in which we took out the outliers who had a reduction in negative symptoms >2SD from the mean and redid the correlation. The result showed that the reductions in the TNF-α levels were still significantly positively associated with PANSS negative subscale reductions (*r* = 0.435, *p* = 0.014). We used the generalized linear mixed model to test the effect of TNF-α on psychopathology before and after treatment, with age, sex, and education level as covariables. Our results showed that the TNF-α levels before treatment were significantly positively correlated with changes in PANSS negative symptoms (β = 0.118, *t* = 2.514, *p* < 0.05).

## Discussion

Our study found that the TNF-α levels in FEDN patients with schizophrenia before treatment were significantly higher than those in healthy controls and chronic patients and that the TNF-α levels before treatment were significantly positively related to changes in PANSS negative symptoms. In chronic patients, the TNF-α levels were negatively related to PANSS general psychopathology symptoms and PANSS total scores. No significant differences were observed in the TNF-α levels between male and female patients with schizophrenia.

We observed that the TNF-α levels in FEDN patients were significantly higher than those in healthy controls and chronic patients, indicating that first-episode patients with schizophrenia may have higher levels of inflammation than healthy controls, consistent with the findings of previous studies [19, 30]. TNF-α increases vascular permeability and promotes the migration of IgG, complement, and effector cells to the infected site; it also promotes retrograde lymph flow into the lymph nodes. Elevation of serum TNF-α levels indicates that the body is in an activated state and that oxidative stress may be present. Activation of cerebral microglia may play a key role in this immune response. When the body encounters various factors leading to peripheral immune dysfunction, inflammatory substances such as cytokines activate brain microglia migration through pathways such as the peripheral nervous system via signaling, secondary messengers, or directly through the blood-brain barrier. Activation of the microglia changes the balance between the excitatory and inhibitory signals of neurons, triggers immune dysfunction in the cerebral cortex, affects neurotransmitters, especially glutamate and aminobutyric acid, and thereby causes psychotic symptoms. This may explain why neuroinflammatory responses play an important role in the etiopathogenesis of schizophrenia [31].

We found that the reduction in the TNF-α levels before and after treatment in FEDN patients was positively related to PANSS negative symptoms and PANSS negative subscale reduction. The TNF-α levels before treatment were significantly positively correlated with changes in PANSS negative symptoms, which is partly consistent with the findings of one study [32], which reported that baseline TNF-α can predict negative symptoms and that inflammatory cytokines may be involved in the development of negative symptoms in some patients. Another study [33] found that the TNF-α levels in chronic patients were positively related to PANSS negative symptoms, especially when the classification of deficit schizophrenia was employed. Traditional antipsychotic drugs are often less effective for negative symptoms in patients with schizophrenia, and negative symptoms seriously affect their quality of life and social functioning [34]. Our findings revealed that inflammatory factors may play a crucial role in the treatment of patients with deficient schizophrenia and that inflammation-related treatments may be innovative therapeutic targets for ameliorating negative symptoms.

Our study found no significant difference in the TNF-α levels in FEDN patients with schizophrenia before and after treatment, consistent with the results of one study [35]. However, Ajami et al. found that the TNF-α levels decreased significantly after treatment [36]. Meta-analyses have also shown different results: TNF-α levels remained constant or decreased after antipsychotic treatment [21, 22]. No significant correlation was found between the TNF-α levels and psychiatric symptoms before treatment in FEDN patients, and there was no significant difference in the TNF-α levels before and after treatment in FEDN patients, which indicated that inflammatory factors may be related to the pathological process of schizophrenia. Inflammatory markers are potentially trait-related in patients with serious mental diseases, which may explain the results of this study. The TNF-α levels in chronic patients were negatively correlated with the PANSS general psychopathology subscales and PANSS total scores in our study, consistent with the findings of one study [23]. Conversely, another study [33] found that the TNF-α levels were positively related to PANSS negative symptoms in chronic deficit patients with schizophrenia. However, another study [37] found that the sTNF-R1 levels were significantly elevated in patients with schizophrenia who were followed up for 1 year, even when there was a reduction in the severity of their psychiatric symptoms. This may be due to the different durations of illness in patients with schizophrenia, different types and dosages of their antipsychotics, discrepancies in treatment response, and differences in the associated somatic diseases.

No sex difference was found in the TNF-α levels in the patients with schizophrenia in our study, which is consistent with the findings of one study [28]. Conversely, an investigation of 91 patients with schizophrenia who took clozapine showed that female patients had higher TNF-α levels than healthy controls; however, no differences were observed in the male patients [38]. The potential biological mechanisms behind this sex difference remain unclear. However, the action of estrogen may be involved. Estrogen has been found to have anti-inflammatory effects. This inflammatory effect is thought to be the link between estrogen and cognitive function [39]. Due to the different bioanalytical materials (e.g., serum, plasma, and cerebrospinal fluid), different research methods and sample sizes, different stages of disease development (e.g., acute, chronic, severe, and alleviative), and confounding factors (e.g., body mass index, smoking, and age) utilized, there are some differences in our results and those of previous studies. Because the correlation between sex and cytokine factors is not clear, sex-matching research will help us to explore possible interactions between sex and inflammatory cytokine levels.

There are some limitations to our study: (1) This study was not a randomized controlled study; thus, there may have been bias in the selection of the sample, and scientific causal inferences cannot be made. In the future, longitudinal studies that are more scientific should be designed with larger sample sizes in order to explore the relationship between TNF-α and psychotic symptoms. (2) The potential effects of various antipsychotics on the TNF-α levels were not assessed in the study. Therefore, future studies should include patients who use different antipsychotics to compare the effects of different drugs on inflammatory factors in each group. (3) Many inflammatory cytokines are involved in the inflammatory process in humans. Different inflammatory factors have mutual effects, and the levels of inflammatory cytokines in different fluids, such as plasma and cerebrospinal fluid, are different. Therefore, in future research, the levels of various inflammatory factors in the cerebrospinal fluid or plasma should be evaluated to more comprehensively explore the potential role and mechanisms of inflammatory factors in the pathogenesis and efficacy of schizophrenia. (4) PANSS has some limitations in assessing the avolition-apathy domain, which is a dimension of negative symptoms. So the Brief Negative Symptom Scale (BNSS) and the Clinical Assessment Interview for Negative Symptoms (CAINS), as well as other scales, may be used to assess the primary and secondary negative symptoms in the future [40].

## Conclusions

Significantly elevated TNF-α levels in FEDN patients and the correlation between TNF-α levels and psychotic symptoms in patients suggest that inflammatory cytokines are involved in the formation and alteration of psychopathic symptoms, especially negative symptoms. At present, the pharmacological mechanism of schizophrenia mostly focuses on neurotransmitters such as dopamine, glutamate, and serotonin. Antipsychotic drugs also improve psychotic symptoms by alleviating the imbalance of these neurotransmitters. However, although the patients received a standardized medical treatment, the effect was still not satisfactory. Some patients had prominent negative symptoms, and their social functioning could not be maintained well. The role of immune functions and neuroinflammatory responses in the pathogenesis of schizophrenia is still worth further investigation, which may provide some targets for the exploitation of new antipsychotic drugs.

## Data Availability

The raw data generated and analyzed in this study are not publically available due to the privacy protection for patients, but the datasets are available from the corresponding author on a reasonable request.
